# Prognostic Significance of HIF-1α Expression in Hepatocellular Carcinoma: A Meta-Analysis

**DOI:** 10.1371/journal.pone.0065753

**Published:** 2013-06-14

**Authors:** Su-Su Zheng, Xiao-Hong Chen, Xin Yin, Bo-Heng Zhang

**Affiliations:** 1 Liver Cancer Institute and Zhongshan Hospital, Fudan University, Shanghai, People’s Republic of China; 2 Center for Evidence Based Medicine, Fudan University, Shanghai, People’s Republic of China; University of North Carolina School of Medicine, United States of America

## Abstract

**Aim:**

Pilot studies have evaluated the correlation between hypoxia-inducible factor-1α (HIF-1α) overexpression and clinical outcome in hepatocellular carcinoma (HCC) patients. However, the results remain inconclusive. To comprehensively and quantitatively summarize the evidence on the suitability of HIF-1α to predict the prognosis of patients with HCC, a meta-analysis was carried out.

**Methods:**

Systematic literature searches were applied to PubMed, Elsevier and Web of Science databases until Feb. 2013. Seven studies (953 patients) were included in this meta-analysis. Pooled measure was calculated from the available data to evaluate the association between tissue -based HIF-1α level and overall survival (OS) and disease-free survival (DFS) in HCC patients. The relation between HIF-1α expression and vascular invasion was also assessed. Data were synthesized with fixed or random effect model, hazard ration (HR) or odds ratio (OR) with its 95% confidence interval (CI) was used as the effect size estimate.

**Result:**

The combined data suggested that HIF-1α overexpression in HCC correlated with poor OS [HR = 1.65 (95% (CI): 1.38, 1.97)] and DFS [*HR* = 2.14 (95% CI: 1.39, 3.29)]. And high HIF-1α expression tended to be associated with vascular invasion [*OR* = 2.21 (95% CI: 1.06, 4.57)].

**Conclusion:**

HIF-1α overexpression indicates a poor prognosis for patients with HCC, it may also have predictive potential for HCC invasion and metastasis.

## Introduction

Hepatocellular carcinoma (HCC) is one of the most common malignant tumors in China. Owing to the improvement of surgical technique and early diagnostic methods, the resection rate of HCC has increased [1].However, the postoperative relapse and metastatic rate remains high [2].Traditional biomarkers, such as Alpha-fetoprotein (AFP) has limited clinical value in predicting prognosis and metastatic recurrence [3].Thus, it is important to identify new molecular predictive markers.

Hypoxia stress is a common phenomenon in human solid tumors and plays a critical role in tumor progression. Under hypoxia condition, hypoxia-inducible factor-1 (HIF-1) is induced and trans-activates many kinds of hypoxia-induced genes. HIF-1 consists of HIF-1α and HIF-1β subunits, HIF-1β is constitutively expressed, while HIF-1α is the functional subunit and is regulated by oxygen. HIF-1α, also known as hypoxia-inducible factor-1α is a hallmark of tumor hypoxia [4]. Increased evidence has revealed that HIF-1α overexpression is well correlated with carcinogenesis and tumor progression in many kinds of cancer. In some malignancies, HIF-1α overexpression was associated with unfavorable prognosis [5], [6]. High expression of HIF-1α was also found in HCC. Recent studies have provided evidence that HIF-1α overexpression plays a role in promoting HCC invasion and metastasis; however, its impact on prognosis in HCC remains controversial [7], [8], [9], [10], [11], [12], [13], [14].

In this paper, we reviewed the currently available evidence in the medical literature on the prognostic significance of HIF-1α expression in HCC to assess the strength of association for better clinical decision-making and further improve patients’ survival for HCC.

## Materials and Methods

### Study Objectives

We primarily aimed at evaluating the prognostic value of HIF-1α expression in tissue-based HCC patients regarding OS and DFS. Our second goal was to assess the association of HIF-1α expression with tumor characteristics, such as vascular invasion, lymphonode metastasis and intrahepatic metastasis.

### Data Sources

Studies were identified by searching PubMed, Elsevier and Web of Science databases. Studies eligible for this analysis was updated on Feb. 2013 using the search terms “HIF-1α”, “hypoxia-inducible factor-1α”, and “HCC”, “hepatocellular carcinoma”, “hepatectomy”, “hepatic tumor”, “hepatic cancer”, “liver cancer”, “liver tumor”, “liver neoplasms”. All eligible studies were retrieved, and their bibliographies were checked for other relevant publications. Additional papers and book chapters were identified by a manual search of the references from the key articles. Searches were limited to clinical trials and papers published in English.

### Criteria for Inclusion and Exclusion

In order to be included, a study had to fulfill the following criteria: (1) patients with distinctive HCC diagnosis by pathology; (2) full length paper with data on survival and HIF-1α expression; (3) the most informative article when multiple articles were published by the same authors or groups.

The following studies were excluded: (1) overlapping articles or duplicate data; (2) articles about cell lines or animals; (3) review articles without original data; (4) conference records; (5) studies lacking information on survival.

### Review of the Studies

Three authors, Su-Su Zheng, Xiao-Hong Chen and Xin Yin independently screened the full text of selected studies to confirm eligibility, assess quality, and extract data. Discrepancies were resolved by discussion and consensus. Study quality was evaluated as suggested by Zhang et al [15]. Each included article, together with its quality score was shown in Table 1.

**Table 1:** Baseline characteristics of the studies in the meta-analysis.

Study(Reference)	Year	Resection	Sample size(M/F, n)	Mean/median age(years)	Vascular invasion(Yes/No, n)	Tumor grade(I–II/III–IV,n)
Huang et al. [9]	2005	Curative	32/4	45.9	24/12	12/24
Wada H et al. [11]	2006	Curative	45/15	63	21/39	33/27
Xie et al. [14]	2008	Resection	59/13	50.57	14/58	55/17
Dai et al. [12]	2009	Curative	95/15	52.4	51/59	58/52
Liu et al. [8]	2010	Radical	169/31	NR	41/159	152/48
Xia et al. [10]	2012	Curative	331/75	51.1	177/229	305/101
Xiang et al. [13]	2012	Curative	61/8	NR	37/32	38/31
**Study quality(Points)**	**HIF-1α Measurement**	**Survival analysis**	**Hazard ratios**	**High HIF-1α** **Definition**	**Number of patients with “high” HIF-1α(n)**	**Number of patients (n)**
4/9	immunohistochemistry	OS	Estimated	positive staining	24	36
6/9	immunohistochemistry	DFS	Reported in text	= 2★	7	60
6/9	immunohistochemistry	OS/DFS	Reported in text	≥10%	37	72
6/9	immunohistochemistry and RT-PCR	OS/DFS	Reported in text	≥10%	39	110
6/9	immunohistochemistry	OS	Estimated	>50%	126	200
5/9	immunohistochemistry	OS	Estimated	≥4▴	212	406
5/9	immunohistochemistry	OS	Reported in text	≥10%	30	69

Vascular invasion was defined as presence of either macro- or microscopic vascular invasion.

Study quality was listed using the results of the Newcastle –Ottawa questionnaire.

M, male; F, female; NR, not reported; OS, overall survival; DFS, disease-free survival; RT-PCR, reverse transcription-polymerase chain reaction; IHC, immunohistochemistry.

★ 2 represented more than 1% nuclear staining and/or with strong cytoplasmic staining.

▴ The final score of each sample was assessed by the sum of the results of the intensity and extent of staining.

### Statistical Methods

Overall survival (OS) was measured from the date of medical treatment (including resection, liver transplantation, etc.) until either the day of death or the day of the last follow-up visit. Disease free survival (DFS) was calculated from the date of treatment to the data when tumor recurrence was diagnosed or to the last visit if recurrence was not diagnosed. Vascular invasion was defined as presence of either macro- or microscopic vascular invasion. The definition of “high” HIF-1α expression was defined by the author (shown in Table 1).

Hazard ratios (HRs), together with corresponding 95% confidence intervals (CIs) were combined across studies. The Parmar method [16] was used to extract data when studies did not have direct information. The strength of association between HIF-1α overexpression and tumor vascular invasion was assessed by odd ratios (OR) with an estimate of 95% CI. A combined HR>1 suggested a higher risk of poor survival, and a synthesized OR>1 predicted a higher incidence of vascular invasion with HIF-1α overexpression. Heterogeneity between trials was checked by the chi-square-based Q-test [17]. A random-effects model was used if a *P*-value of less than 0.10 for the Q-test confirmed heterogeneity across trials [18]. Otherwise, the fixed-effects model was used [19]. Publication bias was assessed by Begg’s funnel plot and Egger’s test [20]. P values <0.05 denoted statistical significance. All of the statistical tests used in our meta-analysis were performed by STATA version 10.0 (Stata Corporation, College Station, TX).

## Results

### 1. Study Characteristics

**Figure 1:**
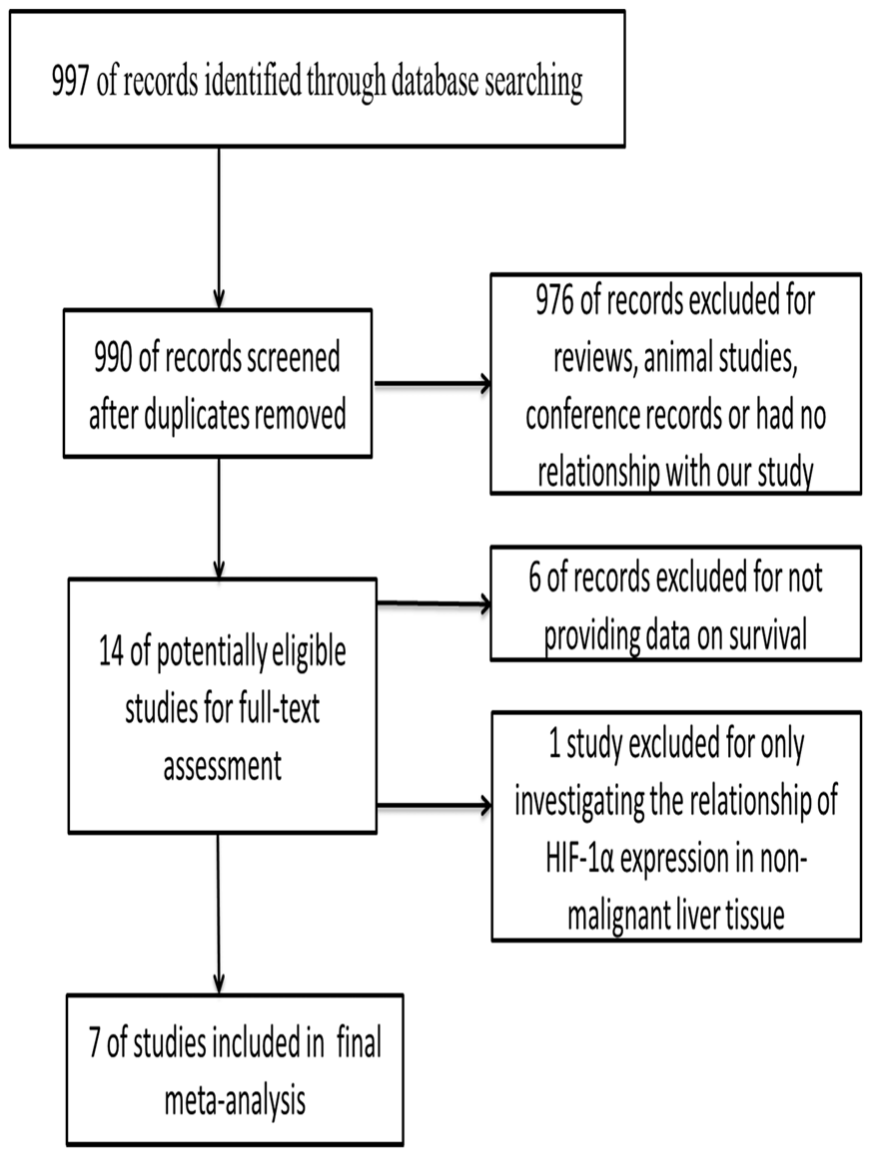
Flow diagram indicating the process of selecting articles for meta-analysis.

Based on the search terms given above, a total of 990 articles were found. After scanning the titles and abstracts, 976 records were excluded for the following reasons: animal studies, reviews, conference records, without data on survival or had no relationship with our analysis. One study was excluded for only investigating the relationship of HIF-1α expression in non-malignant liver tissue. Finally 7 studies [8], [9], [10], [11], [12], [13], [14], published as full papers, were included (Fig. 1). Among the 7 references identified, 3 studies were reported by the same study center [8], [12], [13]; however, each study in one publication was considered separately for pooling analysis, so all of them were assessed in the final meta-analysis. All studies were retrospectively analyzed, 6 of which were done in China [8], [9], [10], [12], [13], [14] and one in Japan [11]. Sample sizes ranged from 36 to 406, and the total number was 953, 475 of whom had HIF-1α overexpression (50%). Of the 7 included studies, 6 provided data on OS, 3 on DFS.

Patients in all studies had surgical resection. Studies reported by Dai et al. [12] and Wada H, Nagano H, Yamamoto H, Yang Y and Kondo M et al. [11], some of the enrolled cases received preoperative antitumor therapy (such as transarterial embolization or transarterial chemoembolization). Another study reported by Xiang et al. [13], all cases went EBRT (external beam radiotherapy) as postoperative adjuvant antitumor therapy. In two studies, all the included patients were Hepatitis B virus (HBV) -related hepatocellular carcinoma [9], [14]. The main characteristics of the studies are described in Tables 1.

### 2. Quantitative Synthesis

#### 2.1 prognosis of HIF-1α expression on prognosis

Owing to a priori assumptions about the likelihood for heterogeneity between primary studies, the pooled OR estimate of the each study was calculated by the random effects model.

**Figure 2:**
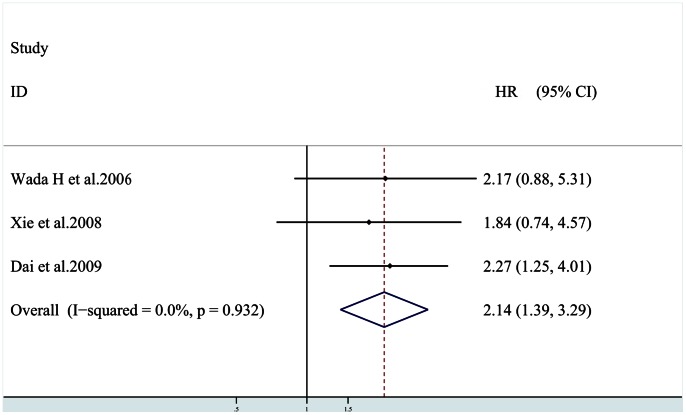
Plot illustrates findings from meta-analysis of the association between HIF-1α overexpression and disease free survival (DFS) in 7 trials. Each study was shown by the name of the first author and the HR with 95% CIs. The summary HR and 95% CIs were also shown (according to the fixed effect estimations).

Three studies [11], [12], [14] provided information on DFS, patients in two studies [11], [12] had preoperative anticancer therapy. The combined data of 3 studies [11], [12], [14] provided information on DFS showed that patients with HIF-1α overexpression had shorter DFS [HR = 2.14 (95% CI: 1.39, 3.29)] (Fig. 2), without any heterogeneity in the data (χ^2^ = 0.14,*Ι* ^2^ = 0.0%,*P* = 0.932). Subgroup analysis indicated that elevated HIF-1α levels were significantly associated with DFS in HCC patients with preoperative adjuvant antitumor therapy [HR = 2.24 (95% CI: 1.37, 3.64)], without any heterogeneity in the data (χ^2^ = 0.14,*Ι* ^2^ = 0.0%,*P* = 0.708).

**Figure 3:**
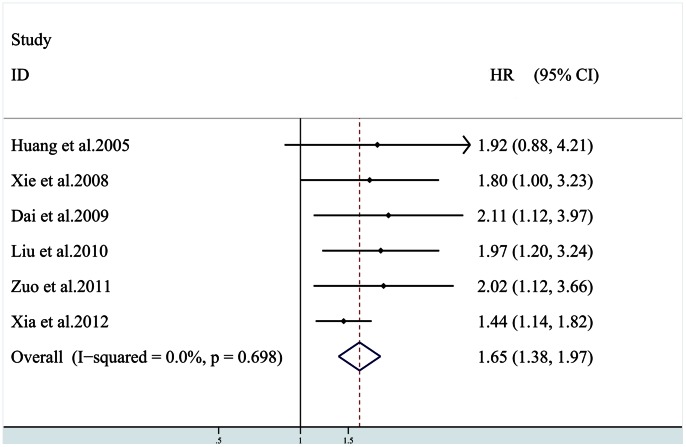
Plot illustrates findings from meta-analysis of the association between HIF-1α overexpression and overall survival (OS) in 7 trials. Each study was shown by the name of the first author and the HR with 95% CIs. The summary HR and 95% CIs were also shown (according to the fixed effect estimations).

6 studies [8], [9], [10], [12], [13], [14] provided data on OS; among the six studies, patients in two studies [9], [10] were all HBV-related hepatocellular carcinoma, and only one study [12] reported that the patients included received preoperative adjuvant antitumor therapy. All six studies investigating OS were pooled into the meta-analysis. Result suggested that HIF-1α overexpression correlates with poor OS [HR = 1.65 (95% CI: 1.38, 1.97)] (Fig. 3), without any heterogeneity in the data (χ^2^ = 3.02,*Ι* ^2^ = 0.0%,*P* = 0.689). Subgroup analysis in HCC patients with preoperative adjuvant antitumor therapy could not be performed for only one study available. In the subgroup analysis by HBV infection, poor OS was also found in HIF-1α overexpression patients [HR = 1.84 (95% CI: 1.15, 2.94)], without any heterogeneity in the data (χ^2^ = 0.02,*Ι* ^2^ = 0.0%,*P* = 0.895).

#### 2.2 Correlation of HIF-1α expression with tumor characteristics

Of the tumor-related factors, it suggested that increased vascular invasion, tumor grade and lymph node metastasis were correlated with HIF-1α according to the 7 selected studies.

**Figure 4:**
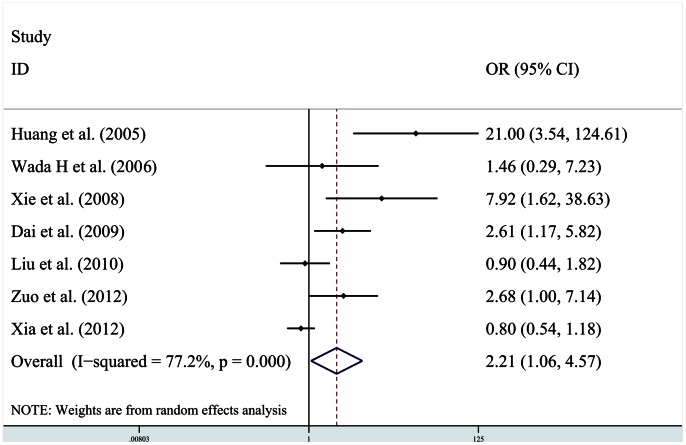
Plot illustrates findings from meta-analysis of the association between HIF-1α overexpression and tumor vascular invasion in 7 trials. Each study was shown by the name of the first author and the OR with 95% CIs. The summary OR and 95% CIs were also shown (according to the random effect estimations).

Data on vascular invasion was shown in all seven studies. Of the seven studies, a statistically significance between HIF-1α expression and vascular invasion was observed in four studies [9], [10], [13], [14].The pooled estimate of correlation between HIF-1α expression and vascular invasion was significant [*OR* = 2.21 (95% CI: 1.06, 4.57)] (Fig. 4), the heterogeneity was evident (χ^2^ = 26.34,*Ι* ^2^ = 77.2%,*P*<0.001).Subgroup analysis showed a trend that elevated HIF-1α levels were associated with vascular invasion in HCC patients with preoperative adjuvant antitumor therapy [OR = 2.32 (95% CI:1.14, 4.74)]. In the subgroup analyses by HBV infection, significant association between HIF-1α overexpression and increased vascular invasion was found [OR = 11.38 (95% CI: 3.40, 38.07)].

All seven studies provided information about tumor grade, but only one study [10] showed statistically significance between HIF-1α overexpression and poor tumor grade. No significant relationship was found after combining the data from the seven studies (data not shown).

Study reported by Xie et al. [14] provided data on the correlation of HIF-1α with lymph node metastasis, however, the pooled analysis could not be performed for no more data from other studies available.

### 3. Publication Bias

**Figure 5:**
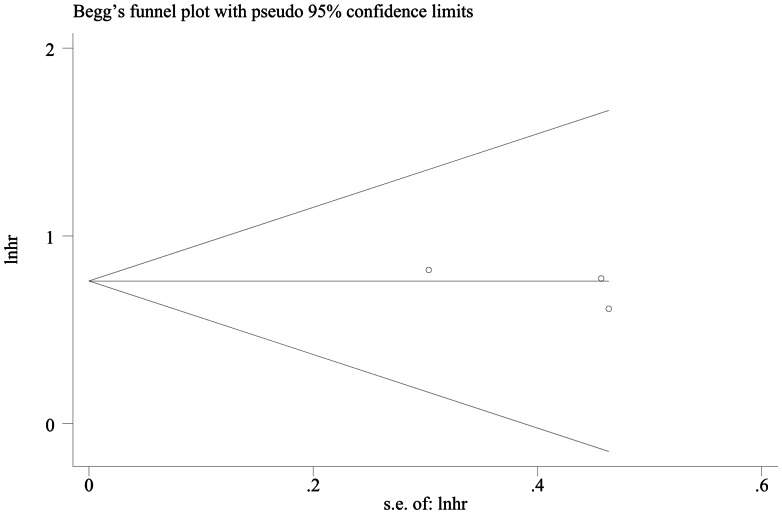
Funnel plot for hazard ratios of recessive model in DFS studies.

**Figure 6:**
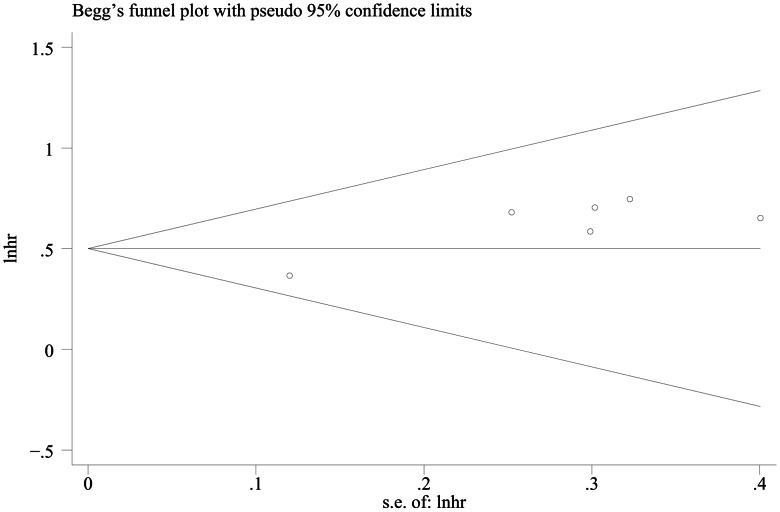
Funnel plot for hazard ratios of recessive model in OS studies.

The results of Egger’s test showed there was no evidence of publication bias on DFS (*P* = 0.445); however, OS publication bias was tested obviously (*P* = 0.008) (Fig. 5, 6).

## Discussion

Identification of the risk of disease recurrence and mortality in HCC patients is critical to guide surveillance and select adjunctive therapies. However, useful biomarkers for predicting prognosis of HCC patients have not been well clarified. Here we introduced a potential clinical useful biomarker: HIF-1α.

Recent studies have demonstrated that HIF-1α is expressed in various human malignancies and HIF-1α overexpression is associated with tumor progression, metastasis potential, treatment failure and increased mortality in many malignancies [21]. In view of its role in regulating tumor pathophysiology, evaluating its prognostic value in HCC is of great clinical importance, which may lead to better patient stratification and targeted therapies in the future.

HCC is characterized by its propensity for hyper-vascularization. Angiogenesis damage to liver blood system in liver cirrhosis and the high proliferation of tumor cells also induces local hypoxia, which was a strong stimulus for HIF-1α [22]. To data, a number of studies have evaluated the association between HIF-1α and clinical outcome in HCC although conflicting data exist [8], [9], [10], [11], [12], [13], [14]. Meta-analysis often searches for an effect not detected by several trials, thus, we performed the meta-analysis to derive a more precise estimation. We believed that the result will provide useful information for decision-making in HCC clinically.

A total of 7 studies, consisting of 953 HCC patients, were included in the final meta-analysis. The pooled results of the meta-analysis showed that high HIF-1α expression correlated with poor DFS and OS in HCC. Subgroup analysis showed that HIF-1α overexpression was also related markedly with DFS and OS in HCC grouped by HBV infection or/and having preoperative adjuvant antitumor therapy.

Present result also demonstrated that HIF-1α overexpression was correlated with increased vascular invasion. The presence of vascular invasion may indicate an increased biological aggressiveness and a greater possibility of systemic diffusion. As shown in previous studies, vascular invasion was the main risk factor for tumor occurrence and had the close relation with tumor invasiveness [21]. So, tumor with HIF-1α overexpression is prone to invasion and metastasis, and associated with poor prognosis. HCCs do not usually express HIF-1α; in this meta-analysis, of 953 HCC patients, 475 (50%) had HIF-1α overexpression. However, once cancer cells acquire HIF-1α expression, they transform to more aggressive and metastatic behavior. Thus, HIF-1α was a poor prognosis factor for HCC patients. It may shed light on novel strategies for the follow-up and HIF-1α can be tested in the selected patients for the better chance of a longer survival.

One thing to be noted here is that prognosis is also influenced by the nature and the activity of the underlying liver disease. Of 7 included studies, 6 were done in hepatitis B virus-endemic area–China, and patients in 2 studies were all HBV-related HCCs. Previous research showed that HIF-1α expression was also found to be significantly correlated with the expression of hepatitis B virus X protein [14]. Thus, there is no certainty that the result performed in the Western world, which mainly include alcoholic patients or patients infected with hepatitis C virus, remain consistent. In the research done in Japan [11], 40 of 60 patients infected with hepatitis C virus. The authors reported that patients with high HIF-1α expression showed worse disease-free survival rates, however, no significant relationship was noted between HIF-1α expression and overall survival rate. So the external validity deserved concerning.

Though we evaluated comprehensively the association between HIF-1α and tumor outcome, several limitations of this meta-analysis also should also be acknowledged. One weakness of our study was publication bias, which could be seen from our publication bias evaluation (especially in the OS studies). It was possibly because positive results were more likely to be published than negative ones. A tendency for journals to only publish positive results leads to the larger magnitude of an association seen in a pooled analysis than it actually is. The second one was heterogeneity. Heterogeneity is a potential problem that may affect the interpretation of the results of all meta-analysis. In our meta-analysis, when investigating the relationship between HIF- 1α expression and tumor vascular invasion, significant heterogeneity was found across the selecting studies. In general, the meta-regression analysis should be conducted. However, the analysis could not be done for there was not enough information. The last one was that the method of detecting HIF-1α expression was not standardized and the assessments of “high” HIF-1α expression were different from authors to authors because of the flexibility of immunohistochemistry. A more precise analysis should be conducted if a more accurate cut-off level is set, such as the serum HIF-1α level.

In conclusion, HIF-1α was correlated with HCC progression and poor prognosis. It could be used as a useful biomarker for predicting tumor invasiveness, metastasis and prognosis of HCC patients. However, the conclusion drawn from our results is hampered by the limitations of the included studies. Future studies evaluating the significance of HIF-1α expression for HCC are strongly recommended.
